# MiR-21 Protected Cardiomyocytes against Doxorubicin-Induced Apoptosis by Targeting BTG2

**DOI:** 10.3390/ijms160714511

**Published:** 2015-06-26

**Authors:** Zhongyi Tong, Bimei Jiang, Yanyang Wu, Yanjuan Liu, Yuanbin Li, Min Gao, Yu Jiang, Qinglan Lv, Xianzhong Xiao

**Affiliations:** 1 Department of Pathology, the Second Xiangya Hospital, Central South University, Changsha 410000, Hunan, China; E-Mail: tongzhongyi2008@163.com; 2 Department of Pathophysiology, Xiangya School of Medicine, Central South University, Changsha 410000, Hunan, China; E-Mails: a15874957966@126.com (Ya.L.); LYB820812@126.com (Yu.L.); gaomin201404@126.com (M.G.); jiangyu146@126.com (Y.J.); lvqinglan2014@163.com (Q.L.); 3 Food Science and Technology College, Hunan Agricultural University, Changsha 410000, Hunan, China; E-Mail: wuyanyang2002@126.com

**Keywords:** microRNA-21 (miR-21), doxorubicin (DOX), cardiotoxicity, B cell translocation gene 2 (BTG-2)

## Abstract

Doxorubicin (DOX) is an anthracycline drug with a wide spectrum of antineoplastic activities. However, it causes cardiac cytotoxicity, and this limits its clinical applications. MicroRNA-21 (miR-21) plays a vital role in regulating cell proliferation and apoptosis. While miR-21 is preferentially expressed in adult cardiomyocytes and involved in cardiac development and heart disease, little is known regarding its biological functions in responding to DOX-induced cardiac cytotoxicity. In this study, the effects of DOX on mouse cardiac function and the expression of miR-21 were examined in both mouse heart tissues and rat H9C2 cardiomyocytes. The results showed that the cardiac functions were more aggravated in chronic DOX injury mice compared with acute DOX-injury mice; DOX treatment significantly increased miR-21 expression in both mouse heart tissue and H9C2 cells. Over-expression of miR-21 attenuated DOX-induced apoptosis in cardiamyocytes whereas knocking down its expression increased DOX-induced apoptosis. These gain- and loss- of function experiments showed that B cell translocation gene 2 (BTG2) was a target of miR-21. The expression of BTG2 was significantly decreased both in myocardium and H9C2 cells treated with DOX. The present study has revealed that miR-21 protects mouse myocardium and H9C2 cells against DOX-induced cardiotoxicity probably by targeting BTG2.

## 1. Introduction

Doxorubicin (DOX), also known as adriamycin commercially, is an anthracycline (ANT) antibiotic first isolated from *Streptomyces peucetius* during the 1960s, together with danorubicin. DOX has been one of the most widely used antitumor drugs. While DOX has been used for successful treatment of several types of cancers, its clinical benefit is limited by the cumulative dose-related cardiotoxicity as a consequence of the lack of specificity, which may ultimately lead to cardiomyopathy and congestive heart failure [1,2,3]. Currently, accumulating evidence indicates that DOX-induced cardiomyopathy is mainly caused by the increased generation of reactive free radicals, which eventually leads to death and apoptosis of cardiomyocytes [4,5,6].Therefore, we speculated that suppression of apoptosis and death might largely rescue DOX-triggered cardiotoxicity.

MicroRNAs (miRNAs) are natural, endogenous, and single-stranded RNA molecules consisting of approximately 22 non-coding nucleotides. They bind to the 3′-untranslated region (UTR) of their target genes and cause the targeted deadenylation and destabilization as well as translational inhibition, thereby influencing the regulation of these genes [7,8]. It has been shown that miRNAs not only play important roles in various human cancers but also are the crucial regulators in cardiac development and pathogenesis of cardiovascular diseases, owing to their involvement in regulating cardiac development, heart function, cardiac hypertrophy and failure [9].

MicroRNA-21 (miR-21) is one of the first identified mammalian miRNAs. Its expression has been found to be activated not only in multiple types of cancers, such as breast, liver, brain, and prostatmyometrial cancers [10], but also in cardiovascular diseases including myocardial infarction and cardiac fibrosis [11,12]. Moreover, recent studies have indicated that miR-21 had a protective effect on ischemia-induced cell apoptosis that was associated with its target gene programmed cell death 4 and activator protein 1 pathway [13]. Furthermore, miR-21 exerted an anti-apoptotic function in ischemia/reperfusion- and hypoxia/reoxygenation-induced cardiocyte apoptosis via the phosphatase and tensin homolog/akt-dependent mechanism [14]. However, the potential benefits of miR-21 action on DOX-induced injury and its underlying mechanism(s) are largely unknown.

B-cell translocation gene 2 (BTG2), also known as PC3 (pheochromocytoma cell 3) or TIS21 (tetradecanoyl phorbol acetate-inducible sequence), belongs to the antiproliferative (APRO) gene family, and is involved in many biological activities in cancer cells [15]. It is an important gene involved in cell differentiation, proliferation, DNA damage repair, and apoptosis in cancer cells. Recent studies have demonstrated that BTG2 is a new target gene of miR-21 in prostate cancer cells, laryngeal cancer cells and melanoma cells. During carcinogenesis, miR-21 plays an important role in regulating BTG2 genes [15]. These BTG2 genes were found to be expressed at high levels in heart and skeletal muscle in both Tong cheng and Landrace pigs [16]. BTG2 regulated necrosis of myocardial cells via inhibiting Akt/Erk, but also activated glycogen synthase kinase 3β (GSK3β) and cyclophilin D in response to H_2_O_2_ [17]. However, it was not clear whether miR-21 protected cardiac myocytes from DOX-induced injury through regulating BTG2.

Therefore, on the basis of these findings, we postulated that miR-21 would protect against DOX-triggered cardiotoxicity. The present study aimed to determine the role of miR-21 in protection of cardiomyocytes against DOX-triggered cardiotoxicity and the underlying mechanisms. Our results demonstrate that miR-21 can alleviate DOX-induced cardiomyocyte apopotosis and further increase cell viability in rat H9C2 cardiomyocytes. Importantly, we found that the mechanism underlying the cardioprotective effects of miR-21 against DOX toxicity is probably mediated through targeting BTG2.

## 2. Results

### 2.1. The Effects of DOX on Cardiac Functions of Mice

**Figure 1:**
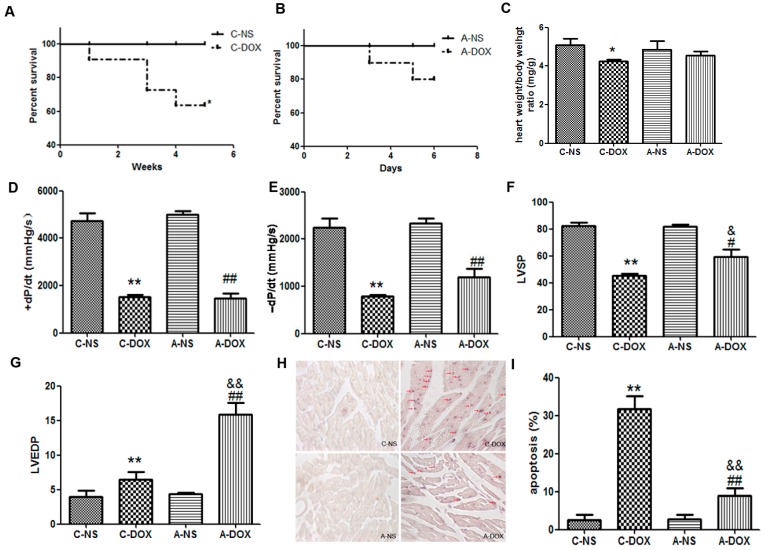
The effects of DOX on the cardiac functions of mice. (**A**). Survival rate of mice in chronic DOX injury group (C-DOX) and chronic normal saline control group (C-NS) groups; (**B**) Survival rate of mice in acute DOX injury group (A-DOX) and acute normal saline control group (A-NS) groups; (**C**) The heart index (the ratio of heart weight to body weight); (**D**) +dP/dtmax, the maximum rate of left ventricular pressure rise; (**E**) −dP/dtmax, the maximum rate of left ventricular pressure decline; (**F**) LVSP, Left ventricular systolic pressure; (**G**) LVEDP, left ventricular end-diastolic pressure; (**H**) Apoptotic myocytes from mouse myocardium were detected by TUNEL staining. TUNEL-positive cells are indicated by violet blues staining in nucleus, and the TUNEL-positive cardiac myocytes are indicated by red arrows; (**I**) Histogram showing the quantitative analysis of TUNEL-positive cells. The values are shown as means ± SD. *, *p* < 0.05, compared to that in C-NS group, *n* = 6; **, *p* < 0.01, compared to that in C-NS group, *n* = 6; ^#^, *p* < 0.05, compared to that in A-NS group, *n* = 6; ^##^, *p* < 0.01, compared to that in A-NS group, *n* = 6; ^&^, *p* < 0.05, compared to that in C-DOX group, *n* = 6; ^&&^, *p* < 0.01, compared to that in C-DOX group, n=6. Note: C-DOX: Chronic DOX injury group. C-NS: Chronic normal saline control group. A-DOX: Acute DOX injury group; and A-NS: Acute normal saline control group.

In order to determine the changes in cardiac functions of the mice in chronic DOX injury group (C-DOX) and acute DOX injury group (A-DOX), the survival rate, heart index (*i.e.*, the ratio of heart weight to body weight), cardiac haemodynamic indices and myocytes apoptosis were accessed. The survival rates were decreased in the C-DOX group compared to those of the A-DOX group (60% *vs.* 80%, Figure 1A,B). We discovered no significant difference in the heart index between the acute normal saline control group (A-NS) and A-DOX groups, but the mice in C-DOX group had a significant decline compared with acute normal saline control group (C-NS) in the heart index (4.241 ± 0.1129 *vs.* 5.076 ± 0.4356, *p* < 0.05, *n* = 6) (Figure 1C). Haemodynamic measurements were recorded to determine the injury effect of DOX on LV function. The DOX-induced changes of LVSP (Left ventricular systolic pressure), LVEDP (left ventricular end-diastolic pressure), +dP/dt (the maximum rate of left ventricular pressure rise), and −dP/dt (the maximum rate of left ventricular pressure decline) were significantly differences both in A-DOX group compared A-NS group and C-DOX group compared with C-NS group (Figure 1D–G). There were significant differences in the LVSP (45.29 ± 1.607 *vs.*59.31 ± 5.605, *p* < 0.01, *n* = 6) and LVDEP (6.454 ± 1.098 *vs.* 15.88 ± 1.719, *p* < 0.01, *n* = 6) between C-DOX and A-DOX groups (Figure 1F,G). Apoptotic myocytes from mouse myocardium were detected by terminal dexynucleotidyl transferase (TdT)-mediated dUTP nick end labeling (TUNEL) staining in figure 1H. The apoptosis cells were significantly increased in C-DOX compared with those in A-DOX (31.67 ± 3.512 *vs.*9.010 ± 2.100, *p* < 0.01, *n* = 6) (Figure 1I). These results suggested the cardiac functions were decreased both in A-DOX and C-DOX, and more aggravated in chronic DOX injury mice than in acute DOX injury mice.

### 2.2. The Effect of DOX Treatment on the Expression of miR-21 in Mice Myocardium and H9C2 Cells

**Figure 2:**
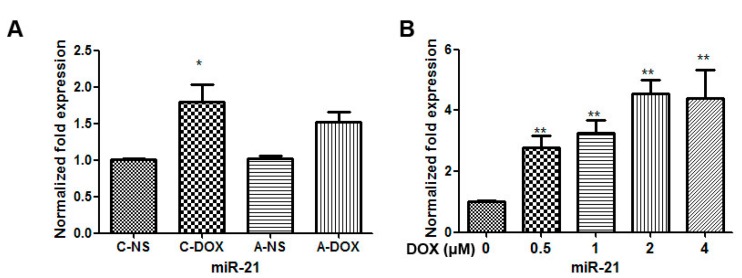
The effects of DOX on the expression of miR-21 in myocardium and H9C2 cells. (**A**) The expression of miR-21 in myocardium. *, *p* < 0.05, compared to that in C-NS group, *n* = 3; Note: C-DOX: Chronic DOX injury group; C-NS: Chronic normal saline control group; A-DOX: Acute DOX injury group; A-NS: Acute normal saline control group; and (**B**) The expression of miR-21 in H9C2 cells treated with various doses (0–4 µM) of DOX for 24 h. **, *p* < 0.01, compared to that at 0 µM DOX, *n* = 3.

To determine whether miR-21 is involved in mediating DOX-induced cardiotoxicity, the changes in the levels of its miRNAs were measured by quantificational real-time polymerase chain reaction (qRT-PCR). As shown in Figure 2A, the expression level of miR-21 in C-DOX group was significantly increased to 1.8-fold as compared with that in C-NS group (1.800 ± 0.4078 *vs.* 1.003 ± 0.03512, *p* < 0.05, *n* = 3). The expression level of miR-21 in A-DOX group was no significant difference compared with that in A-NS group (1.520 ± 0.2307 *vs.*1.017 ± 0.07638, *p* > 0.05, *n* = 3). As shown in Figure 2B, exposure of cardiac myocytes to DOX at doses ranging from 0–4 µM for 24 h resulted in a significantly dose-dependent increase in miR-21 expression level in H9C2 cells (*p* < 0.01, *n* = 3).

### 2.3. The Effects of miR-21 Mimics and Inhibitors on the Injury Mediated by DOX in H9C2 Cells

**Figure 3:**
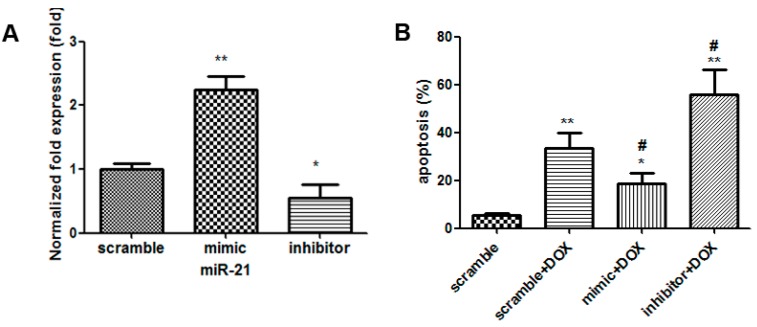

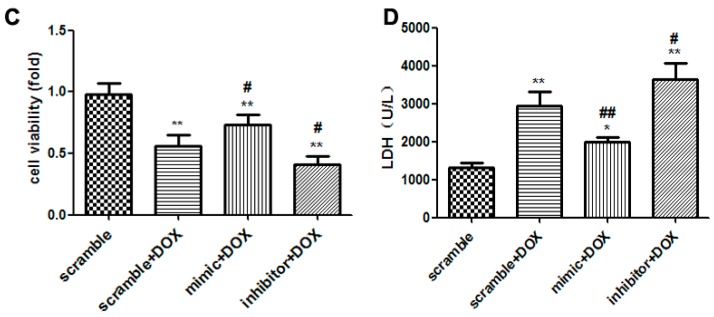
Overexpression and knockdown of miR-21 influenced the DOX-induced cytotoxicity in H9C2 cells. (**A**) The levels of miR-21 in H9C2 cells transfected with rno-miR-21 mimics/inhibitors/scrambled controls were measured by qRT-PCR. *, *p* < 0.05, compared to that of cells treated with scramble, *n* = 3; **, *p* < 0.01, compared to that of cells transfected with scramble, *n* = 3; (**B**) Apoptosis induced by DOX (1 µM for 24 h) in H9C2 cells transfected with miR-21 mimics/inhibitors. *, *p* < 0.05, compared to that of cells transfected with scramble, *n* = 3; **, *p* < 0.01, compared to that of cells transfected with scramble, *n* = 3; ^#^, *p* < 0.05, compared to that of cells transfected with scramble + DOX, *n* = 3; (**C**) Cell viability; and (**D**) Activity of lactate dehydrogenase. *, *p* < 0.05, compared to that of cells transfected with scramble, *n* = 6; **, *p* < 0.01, compared to that of cells transfected with scramble, *n* = 6; ^#^, *p* < 0.05, compared to that of cells transfected with scramble + DOX, *n* = 6; ^##^, compared to that of cells transfected with scramble + DOX, *p* < 0.01, *n* = 6.

To evaluate the potential roles of miR-21 in DOX (1 µM for 24 h)-induced cardiotoxicity, cell apoptosis, cell viability and release of lactate dehydrogenase (LDH) were measured in H9C2 cells by the gain- and loss- of -function approaches with a miR-21 mimic/inhibitor. Compared with that in the cells treated with scramble, the expression level of miR-21 was increased to 2.17-fold in H9C2 cells transfected with rno-miR-21 mimics (*p* < 0.01, *n* = 3); Compared with that in the cells treated with scramble, the expression levels of miR-21 was decreased to 0.55 folds in H9C2 cells transfected with a rno-miR-21 inhibitor (*p* < 0.05, *n* = 3) (Figure 3A). Compared with that in the cells treated with the scramble + DOX, apoptotic rate was decreased from 37% ± 2.1% to 18% ± 2.8% in cells treated with miR-21 mimics(*p* < 0.05, *n* = 3), but the percentage of the apoptotic cardiac myocytes was increased to 57% ± 4.3% in the cells treated with miR-21 inhibitor (*p* < 0.05, *n* = 3) (Figure 3B). Cell viability assay and LDH measurement showed that overexpression of miR-21 was sufficient to cause the increase in cell viability (*p* < 0.05, *n* = 6) and reduce the release of LDH (*p* < 0.01, *n* = 6). Knocking down miR-21 obviously decreased the cell viability (*p* < 0.05, *n* = 6) and obviously increased the release of LDH (*p* < 0.05, *n* = 6) (Figure 3C,D). These results suggest that miR-21 attenuates DOX-induced injury on cardiac myocytes, whereas inhibition of its expression may aggravate DOX-induced apoptosis of cardiac myocyte.

### 2.4. miR-21Regulatedthe Expression of BTG-2 in H9C2 Cells

**Figure 4:**
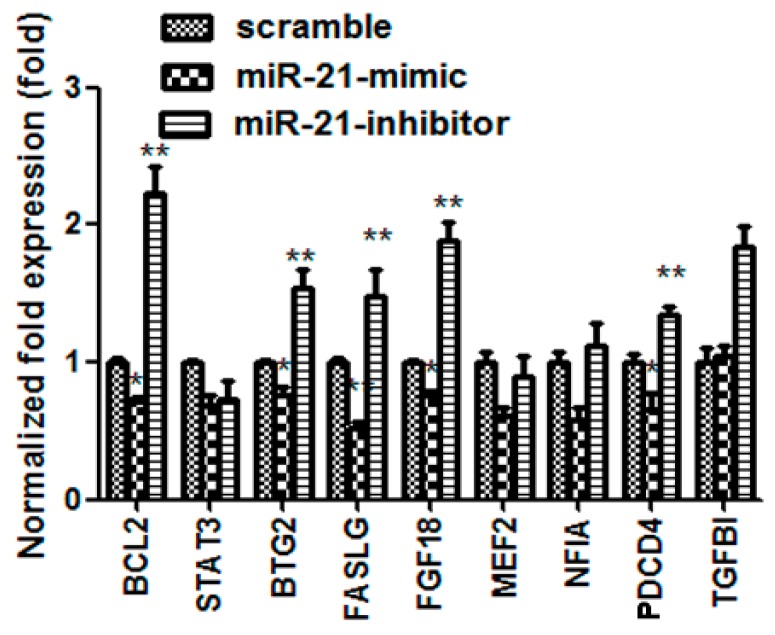
The expression of target mRNAs of miR-21 were tested by qRT-PCR. BCL2, B-cell CLL/lymphoma 2; STAT3, signal transducer and activator of transcription 3; BTG2, B cell translocation gene 2; FASLG, Fas ligand; FGF18, fibroblast growth factor 18; MEF2, myocyte enhancer factor 2C; NFIA, nuclear factor I/A; PDCD4, programmed cell death 4; TGFBI, transforming growth factor, β-induced. *, *p* < 0.05, compared with scramble, *n* = 3; **, *p* < 0.01, compared with scramble, *n* = 3.

**Figure 5:**
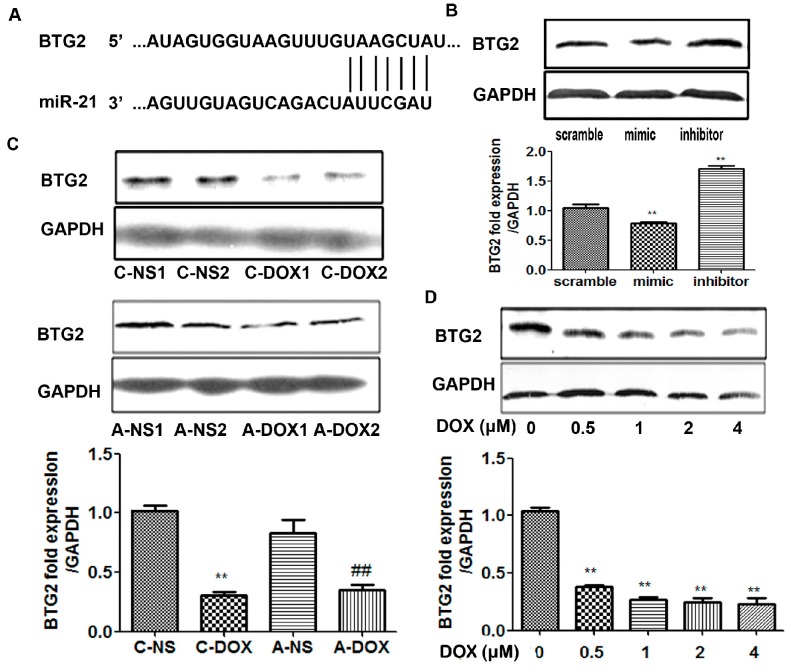
The effects of DOX on the expression of BTG-2 in myocardium and H9C2 cells. (**A**) Predicted consequential pairing of BTG2 (**top**) and miR-21 (**bottom**) by TargetScan; (**B**) The expression levels of BTG2 in H9C2 cells transfected with rno-miR-21 mimics/inhibitors/scrambled controls detected by western blotting. **, *p* < 0.01, compared to that of cells transfected with scramble, *n* = 3; (**C**) The expression levels of BTG2 in A-DOX and C-DOX myocardium. A-NS1, A-NS2 represented two different samples. C-DOX: Chronic DOX injury group; C-NS: Chronic normal saline control group. A-DOX: Acute DOX injury group; A-NS: Acute normal saline control group. 1, 2 behind them represent two different samples. **, *p* < 0.01, compared to that of C-NS group, *n* = 3; ^##^, *p* < 0.01, compared to that of A-NS group, *n* = 3; and (**D**) The expression levels of BTG2 in H9C2 cells treated with DOX (0–4 µm for 24 h). The panel above was the representative western blotting image and the panel below was quantitative analysis. **, *p* < 0.01, compared to that at 0 µM DOX, *n* = 3.

MicroRNAs (miRNAs) are involved in negatively regulating gene expression via degradation or translational inhibition of their target mRNAs. We used bioinformatics software (TargetScan or PicTar) to predict 9 biological targets of miR-21. The results showed that BCL-2, BTG2, FGF18, PDCD4, FASLG may be the biological targets of miR-21 by the gain- and loss- of-function approaches with a miR-21 mimic and an inhibitor (Figure 4). Prior to this study, it was not clear whether BTG2 was the true target of miR-21 in cardiac myocytes and whether miR-21 protected cardiac myocytes from DOX-induced injury through regulating BTG2, so we have chosen BTG2 for further study. The results in Figure 5A showed the predicted consequential pairing of BTG2 (top) and miR-21 (bottom) (http://www.targetscan.org/). miR-21 overexpression significantly inhibited BTG2 protein expression (*p* < 0.01, *n* = 3) whereas knock-down miR-21 expression obviously increased BTG2 protein expression (*p* < 0.01, *n* = 3) (Figure 5B). Then, the expression levels of BTG2 in myocardium and H9C2 cells treated with DOX were examined by western blotting. The results showed that the expression of BTG2 was significantly decreased both in A-DOX (*p* < 0.01, *n* = 3) and C-DOX groups (*p* < 0.01, *n* = 3) (Figure 5C). The protein level of BTG2 was also decreased in H9C2 cells treated with different doses (0.5–4 µM) of DOX (*p* < 0.01, *n* = 3) (Figure 5D). These results suggest that BTG2 is target gene of miR-21 in cardiac myocytes and that miR-21 may protect cardiacmyocytes from DOX-induced injury through post-transcriptional regulation of BTG2.

## 3. Discussion

A number of studies have demonstrated that DOX is one of the most widely and successfully used antitumor drugs. Despite of its well therapeutic effects, its clinical applications are still limited because of its cumulative and dose-dependent cardiac toxicity [1,2,3]. In fact, multiple intravenous DOX treatments over a period of several months were found to result in the development of cardiomyopathy and congestive heart failure (CHF) in humans [18]. At present, two kinds of methods have been used to establish the DOX-induced injury mouse models: (1) Acute DOX injury model: DOX was administered by intraperitoneal (ip) injection at one dose of 20 mg/kg [19] or a daily dose of 5 mg/kg to a cumulative amount of 20 mg/kg [20]; (2) Chronic DOX injury model: DOX was administered by intraperitoneal (ip) injection at a weekly dose of 3–4 mg/kg to a cumulative amount of 20 mg/kg [19] or a weekly dose of 6 mg/kg for a total of four weeks [21]. In order to compare which mouse model is matched more closely with clinical status, we used both the acute DOX injury groups and chronic DOX injury models, and found that DOX remarkably depressed LV-function, decreased heart indices (the ratio of heart weight to body weight) in mice in both A-DOX and C-DOX groups. However, compared to those in the A-DOX group, the cardiac injury was aggravated more seriously in the C-DOX group (Figure 1). Therefore, chronic model of DOX-induced cardiotoxicity can be more closely resemble to the clinical patients who are typically receive lower levels of DOX given over many weeks [18]. This model is more suitable for the future scientific research.

In cancers, miRNAs-21 is one of the most important miRNAs and is highly expressed in many solid tumors where it is involved in cell proliferation, differentiation and apoptosis, and thus, it is closely related to tumors growth, invasion, and metastasis [22]. Thus, targeting miR-21 and inhibiting its activity may be emerging as a promising therapeutic option and offers a potential new mode of cancer therapy. For example, it has been demonstrated that combining both DXR (doxorubicin) and miR-21i (inhibitor) significantly reduced tumor cell proliferation, invasion and migration *in vitro*, and that combining both DXR and miR-21i is a new strategy for the therapy of glioblastoma [23]. Recent studies have indicated that miR-21 has a protective effect on ischemia-induced cell apoptosis associated with its target genes, such as PDCD4 and PTEN (phosphatase and tensin homolog) [13,14]. In renal I/R injury, miR-21 is more likely a double-edged sword, which has both protective and pathological roles [24]. However, the effect of miR-21 on DOX induced cardiotoxicity is not clear.

To further define the potential role of miR-21 in mediating DOX-induced cardiotoxicity, miR-21 expression was modulated by using both miR-21 mimic and inhibitor, respectively. Interestingly, up-regulation of miR-21 expression via miR-21 mimic inhibited DOX-induced apoptosis and the release of LDH. In contrast, DOX-induced apoptosis and the release of LDH were exacerbated after down-regulation of miR-21 expression via miR-21 inhibitor. These results clearly suggest that miR-21 has anti-apoptotic and anti-cell death effects against DOX-induced cardiotoxicity (Figure 3). However, the study by Tong’s group reported that miR-21 promoted cardiac fibrosis and development of heart failure with preserved left ventricular ejection fraction by up-regulating BCL-2 [25]. We speculated that this could be due to the face that miR-21 exerted its biological functions through different signaling pathways under different circumstances.

Regarding how miR-21 protected cardiac myocytes from DOX-induced injury, a bioinformatic analysis suggests that BTG2 may be a potential target of miR-21. BTG2 has been known to be involved in the regulation of the proliferation of neural progenitor cells during neurogenesis [26]. The overexpression of BTG2 may inhibit the growth and proliferation through inhibiting the protein expression of cyclin D1, and invasiveness through inhibiting the protein expression of MMP-1 (matrix metalloproteinase-1) and MMP-2 (matrix metalloproteinase-2) in the A549 human lung cancer cell line [27,28]. Moreover, the regulation of BTG2 by miR-21 has been reported in human laryngeal carcinoma [29]. Thus, the expression of BTG2 was examined in myocardium and H9C2. The results firstly confirmed that DOX decreased the expression of BTG2 protein in both myocardium and H9C2 cells (Figure 5). Our next plan is to further elucidate how miR-21 regulates BTG2 expression and what precise role it plays in mediating DOX-induced apoptosis of cardiomyocytes.

It is worth noting that our recent study with a transgenic model of myocardial overexpression of nucleolin revealed that miR-21 was one of eleven miRNAs whose expression was up-regulated by nucleolin (Jiang *et al.*, 2015, unpublished data). In another published study, we found that nucleolin protected the heart from ischemia-reperfusion injury [30]. More recently, Monte *et al.* conducted a systematic proteomics of cardiac chromatin and identified nucleolin as a regulator of growth and cellular plasticity in cardiomyocytes [31]. In this study, we found that miR-21 regulated the expression of BTG2. Taking together, these studies have identified important connections of nucleolin-miR21-BTG2. Thus, it would be interesting and important to further investigate the detailed molecular components of these connections and their roles in regulation of the growth and other cellular processes in cardiomyocytes.

In summary, in this study, we have documented that miR-21 is a potent protector for cardiomyocytes against DOX-induced cardiotoxicity probably via regulating the expression of BTG2, a member of an anti-proliferative gene family. Further studies are needed to investigate their functional roles in regulation of the growth and other cellular processes of cardiomyocytes.

## 4. Materials and Methods

### 4.1. Animals and Treatment

Male wild-type Balb/c mice, 20 ± 2.1 g, were purchased from the Animal Center, Central South University (Changsha, China). The mice were housed in a temperature-and humidity-controlled animal house at 25 °C with a 12:12 h day/night cycle. The mice with similar body weight were divided randomly into four groups with 10 mice in each group and treated as follows: (1) Acute DOX injury group (A-DOX): Mice in this group were injected intraperitoneally (i.p.) with DOX (Sigma-Aldrich Co., St. Louis, MO, USA) diluted in pyrogen-free normal saline at 4 mg/kg/day and continuously injected for five days with a cumulative dose of 20 mg/kg; (2) Acute normal saline control group (A-NS): mice in this group were injected with normal saline and used as the control. The two groups of mice were euthanized on the sixth day; (3) Chronic DOX injury group (C-DOX): Mice in this group were injected i.p. with DOX diluted in pyrogen-free normal saline at 5 mg/kg/week and continuously injected for four weeks with a cumulative dose of 20 mg/kg; (4) Chronic normal saline control group (C-NS), the mice that were injected with normal saline were used as the control. The two groups of mice were euthanized on the 29th days. Protocols for animal breeding and use for the experiments were approved by the Medical Ethics Committee of Xiangya Hospital, Central South University (No. 201402027).

### 4.2. Cardiac Haemodynamic Measurements

Cardiac hemodynamic measurements were conducted on Day 29 and 7 days after the last DOX administration in the chronic DOX model and conducted on day 6 in the acute DOX model according to a method described in our previous work [32]. In brief, the mice were anaesthetized via i.p. injections with 10% chloral hydrate (2.5 mL/kg) and placed on the controlled heating pads. The core temperature, measured via a rectal probe, was maintained at 36–38 °C. A small cannula filled with heparin saline (500 U/mL) was inserted into the left ventricle through the apex with the chest open and mechanically ventilated, and positioned along the cardiac longitudinal axis. After stabilization for 2 min, the pressure signal was continuously recorded using a MacLab A/D converter (AD Instruments, Mountain View, CA, USA). The left ventricular systolic and end-diastolic pressures were measured. The maximal slope of systolic pressure increment (+dP/dt) and the maximum rate of left ventricular pressure decline (−dP/dt) were calculated. After the haemodynamic measurements were made, the mice were euthanized by cervical dislocation.

### 4.3. Cell Culture

H9C2 cells, subclones of the original clonal cell line derived from embryonic BD1X (strain) rat heart tissue, were purchased from the Shanghai Cell Bank of the Chinese Academy of Sciences (Shanghai, China) and routinely grown in Dulbecco’s Modified Eagle (DMEM) medium supplemented with 10% fetal bovine serum at 37 °C under 5% CO_2_. The cells were exposed to DOX at the concentration range of 0–4 µM for 24h.

### 4.4. Transfection Experiments

H9C2 cells at 80% confluence were transfected with rno-miR-21 mimics (5′-UAGCUUAUCAGACUGAUGUUGA-3′) or rno-miR-21 inhibitors (5′-UAGCUUAUCAGACUGAUGUUGA-3′), or scrambled controls (Qiagen, Cambridge, MA, USA), respectively, at a final concentration of 20 μM with the use of hiperfect transfect reagent (Qiagen). At 48 h after transfection, the cells were harvested for further study.

### 4.5. RNA Isolation and Quantitative Real-Time PCR Analysis

Total RNA was isolated from myocardium and H9C2 with the miRNeasy mini kit (Qiagen). The miScript reverse transcription kit (Qiagen) and miScript SYBR Green PCR kit (Qiagen) were used to measure the expression levels of miR-21 with a model 7500 Fast Real-Time PCR system (Applied Biosystems, Foster City, CA, USA), with a miR-21-specific primers and the miScript Universal Primer (Qiagen). Expression level of the U6B small nuclear RNA (RNU6B) was used as the endogenous control to normalize the sample data. Relative expression levels were calculated with the 2^–^^ΔΔ*C*t^ method. Each of the experiments were repeated at least three times.

### 4.6. Protein Preparation and Western Blotting

Proteins were extracted from mouse myocardium and H9C2 cells with radio immunoprecipitation assay (RIPA) lysis buffer. Protein extracts were centrifuged at 12,000× *g* for 15 min. The protein concentration was measured with BCA measurement. Total protein (10–30 μg per lane) was separated on a 15% or 12% sodium dodecyl sulfate-polyacrylamide gel electrophoresis (SDS-PAGE) and transferred to a polyvinylidene fluorid emembrane (Millipore, Billerica, MA, USA). The membrane was soaked in 5% bovine serum albumin in Tris-buffered saline Tween (TBST, pH 7.6). The membrane was then incubated overnight at 4 °C with a rabbit polyclonal antibody against BTG2 (BBI, Boston biomedical Inc., Cambridge, MA, USA) at a dilution of 1:200, or with a mouse monoclonal antibody against β-actin (Sigma-Aldrich) at a dilution of 1:1000. Subsequently, the membrane was rinsed with tris-buffered saline and tween 20 (TBST) and incubated with horseradish peroxidase-conjugated goat anti rabbit or mouse immunoglobulin G (IgG) antibody for 1 h on a rotating platform at room temperature. The membrane was then washed with TBST three times and incubated with diaminobenzidine (DAB) chromagen for color development. The experiments were repeated at least three times.

### 4.7. Assays of Cell Viability and Activities of Lactate Dehydrogenase

Cell viability was assayed using 3-(4,5)-dimethylthiahiazo (-z-y1)-3,5-di-phenytetrazoliumromide (MTT) cell proliferation and cytotoxicity assay kit (Beyotime Institute of Biotechnology, Shanghai, China) according to the manufacturer’s recommendations.The activities of LDH were determined with a LDH assay kit (Nanjing Jiancheng Biotechnology Institute, Nanjing, China) according to the manufacturer’s instructions and expressed as units per liter. The results were expressed as means ± standard deviation (SD) of six separate samples (*N* = 6).

### 4.8. Apoptosis Assessment by TUNEL Assay

To detect apoptosis, TUNEL staining was performed according to the following instructions. Paraffin embedded sections of samples were deparaffinated and hydrated, and then incubated in 20 µg/mL protease K at room temperature for 15 min. After being washed with phosphate-buffered saline (PBS) thrice, the samples were put into TDT buffer (0.14 M Na-Cacodylateand 1 mM Cobalt Chloride + 1.25 mg/mL BSA) for 10 min. After being washed with PBS for 5 min thrice, 140 mL of TUNEL reaction mixture (digoxigenin-11-dUTP 1 nmol + Ttase 400 U into 1 mL TDT buffer) was added to the sample, and incubated at 37 °C for 3 h. After being washed 2× in saline sodium citrate for 15 min, 0.5× saline sodium citrate for 10 min, 0.1× saline sodium citrate for 10 min, 1% bovine serum albumin in Tris buffer incubated for 30–60 min, the sections were incubated with anti- digoxigenin -AP (1:1000, Roche Applied Science, Indianapolis, IN, USA) for 2 h. After being washed with Tris buffer for 15 min twice, chromogenic reagent (NBT (4-nitro blue tetrazolium chioride, Roche Applied Science, Indianapolis, IN, USA) 4.5 µL + BCLP (5-bromo-4-chloro-3-indoyl-phosphate4-touidine salt, Roche Applied Science, Indianapolis, IN, USA) 3.5 µL in 7 mL Tris buffer) was added. Chromogenic reaction was stopped when the apoptotic nuclei showed purplish blue. For each myocardial specimen, tissue sections were examined microscopically at ×200 magnification and 10 randomly selected fields persection were counted. The percentage of apoptotic cells was calculated by the apoptotic index, *i.e.*, dividing the number of positive-staining myocyte nuclei by the total number of myocytes.

### 4.9. Flow Cytometry

Flow cytometry was performed using Annexin-V-FLUOS Staining Kit (Roche Diagnostics GmbH, Roche Applied Science 6298, Mannheim, Germany) according to instructions provided in the kit. The cells were analyzed on a flow cytometer (Beckman Institute For Advanced Science and Technology, Urbana, IL, USA) using 488 nm excitation and a 515 nm band pass filter for fluorescein detection and a filter >600 nm for PI detection. The results were expressed as means ± SD of three separate samples.

### 4.10. Computational Prediction of miRNA Target Genes

MicroRNA-21 target genes in mouse were screened by merging the results of computational prediction algorithms provided at the TargetScan (http://www.targetscan.org) and PicTar (http://pictar.bio.nyu.edu).

### 4.11. Statistical Analysis

The data were analyzed with the SPSS16.0 version. The data were expressed as mean ± SD. Differences between two groups were analyzed by unpaired Student’s *t*-test. Differences between three or more groups were analyzed by statistical analysis using one-way ANOVA (analysis of variance) followed by Student-Newman-Keuls. Mortality comparisons were conducted by Kaplan-Meier survival curve analysis. The logrank test was used to assess survival differences. A value of *p* < 0.05 was considered statistically significant.

## 5. Conclusions

In summary, in this study, we have documented that miR-21 is a potent protector for cardiomyocytes against DOX-induced cardiotoxicity probably via regulating the expression of BTG2, a member of an anti-proliferative gene family. Further studies are needed to investigate their functional roles in regulation of the growth and other cellular processes of cardiomyocytes.
